# Reply to Palumbo et al. Comment on “Flatscher et al. Impact of Mediterranean Diet on Lipid Composition in the Colaus-PsyColaus Study. *Nutrients* 2023, *15*, 4659”

**DOI:** 10.3390/nu16101425

**Published:** 2024-05-09

**Authors:** Mélisande Flatscher, Antoine Garnier, Pedro Marques-Vidal, Vanessa Kraege

**Affiliations:** 1 Faculty of Biology and Medicine, Lausanne University, 1011 Lausanne, Switzerland; 2 Department of Internal Medicine and Specialties, Fribourg Hospital, 1708 Fribourg, Switzerland; antoine.garnier@h-fr.ch; 3 Department of Medicine, Internal Medicine Division, Lausanne University Hospital, 1011 Lausanne, Switzerland; pedro-manuel.marques-vidal@chuv.ch; 4 Innovation and Clinical Research Directorate, Lausanne University Hospital, 1011 Lausanne, Switzerland; vanessa.kraege@chuv.ch; 5 Medical Directorate, Lausanne University Hospital, 1011 Lausanne, Switzerland

We thank the authors of the commentary [1] for their analysis of our study [2] and their suggestions in response to its limitations which may help to improve future studies on this subject.

One of the main limitations of the prospective part of our study is indeed the assessment of Mediterranean diet (MD) adherence at the first follow-up only, whereas changes in lipids and incidence of dyslipidemia were measured over time. 

Regarding the Vormund score, although it has not been validated, this adapted MD score has shown an inverse association between MD and cardiovascular and cancer mortality in the Swiss population [3]. Moreover, in our study, it is interesting to note that the application of two different scores (Vormund and Trichopoulou) shows similar results.

The semi-quantitative food frequency questionnaire (FFQ) has its limitations. A more accurate method with higher-quality nutritional data, such as the dietary record (DR), could be an excellent alternative. Still, its implementation in large epidemiological studies with thousands of participants is difficult and very costly, as it requires more resources and considerable involvement from participants. Further, it could lead to selection bias, as only the most motivated participants would complete the records. As mentioned, new technologies could solve most of these difficulties for future epidemiological studies [4]. Another difficulty could be the application of reference scores measuring MD adherence on a DR, such as the Trichopoulou score, as these have been used mainly on FFQs.

Finally, we agree that our study contradicts previous literature on the beneficial effect of MD on lipid profile [5,6]. However, contradiction is part of the scientific methodology, and not all results have to be consistent with existing literature. The proportion of studies carried out in northern countries remains a minority compared with those carried out in the Mediterranean area. We therefore confirm the need for more reliable tools to measure nutrition and the need to adapt diets to the foods available in each region, to obtain the best benefit on lipids.
